# An Evaluation of Different Parameters to Screen Ornamental Shrubs for Salt Spray Tolerance

**DOI:** 10.3390/biology9090250

**Published:** 2020-08-27

**Authors:** Stefania Toscano, Ferdinando Branca, Daniela Romano, Antonio Ferrante

**Affiliations:** 1 Department of Agriculture, Food and Environment (Di3A), Università degli Studi di Catania, Via Valdisavoia 5, 95123 Catania, Italy; stefania.toscano@unict.it (S.T.); fbranca@unict.it (F.B.); 2 Department of Agricultural and Environmental Sciences, Università degli Studi di Milano, Via Celoria 2, 1-20133 Milan, Italy; antonio.ferrante@unimi.it

**Keywords:** Mediterranean area, marine aerosol, species selection, photosynthesis, chlorophyll *a* fluorescence

## Abstract

In the context of seaside landscaping, one of the greatest challenges for practitioners and scientists is to select suitable species that are able to tolerate salt spray. This is the key aspect for a wide number of potentially suitable species. The objectives of this study were (1) to identify plant traits associated with species tolerance to salt stress and (2) to evaluate the responses of different shrub species to salt spray. For this purpose, a study was conducted to determine the effects of salt spray on twenty-four ornamental shrubs using rapid and easy-to-use methodology. The species were subjected twice a week to nebulization treatment with simulated seawater solution for 60 days. Every 20 days, net photosynthesis, stomatal conductance, transpiration rate, and chlorophyll *a* fluorescence were determined. Furthermore, dry biomass of the different organographic portions, leaf number, leaf area, Specific Leaf Area, chlorophyll content, and leaf damage were determined at the end of the experiment. The species exposed to seawater solution showed different physiological and morphological responses. Based on the above indices, these ornamental shrubs were screened and categorized as tolerant, moderately tolerant, or susceptible. The results suggest that Convolvulus, Ceratonia, and Ligustrum are more tolerant to salt spray than numerous other genotypes; L. langmaniae, Cascabela, and L. frutescens, conversely, are more sensitive. Among the plant traits, the morphological parameters thoroughly characterized the effects of the salt spray, but they were destructive, with the only exception being the leaf damage percentage. This last non-destructive parameter is interesting considering the aesthetic value that ornamental plants must have. The physiological parameters, and in particular photosynthesis activity, can instead be used as a non-destructive screening method to select species suitable for ornamental green spaces near the sea.

## 1. Introduction

In coastal areas, abiotic stresses that adversely affect plant growth and development are represented by soil salinity, salt spray [1,2,3,4,5], and deposition of salt on leaves [6]. Salt spray, in particular, is formed by seawater droplets carried to land by the wind. Salt may enter the aerial organs of plants, especially where small surface injuries are present [1].

Plants near the sea have adapted to survive direct contact with salt on their leaves, although exposure to marine aerosol may reduce or inhibit their growth [7,8] and reduce their ornamental value [5]. The effects of salt mainly depend on the intensity and length of exposure [9]. However, most studies have focused on the response of plants to saline irrigation [7,10,11,12], and less attention has been devoted to their response to marine aerosol when irrigated with non-saline water.

Plants are more sensitive to damage when aerosolized saline water is deposited directly on the leaves than when saline water is distributed to the soil and roots [13,14]. However, the response to salt has rarely been analyzed when salt water is applied directly to the leaves and not distributed in the soil. Tolerance to salt stress is due to the morphological characteristics of the leaves. The thickness of the epidermis layer, associated with the presence of trichomes, can help to reduce the effects of salt damage, preventing salt from directly coming in contact with the leaf surface. In plants of *Agrostis stolonifera* [15] and *Festuca rubra* [16] subjected to marine aerosol, the wettability characteristics and morphology of the leaves were shown to influence the absorption of sodium. Salt spray tolerance depends on the characteristics of the structures that protect the leaf, such as the thickness of the cuticle [17]. A thick leaf cuticle, able to reduce transpiration, only in part prevents ions from penetrating the leaves and the consequent osmotic and ionic stress. The damage determined by the ion concentration, especially Na^+^ accumulation [6], is associated with water stress, disrupts membranes and enzyme systems [18], inhibits the uptake of nutrients [19], causes necrosis or loss of leaves, and can lead to mortality [20].

Toxic ions are absorbed through the stomata and the cuticle, causing deterioration of the waxy cuticles and alteration in the cell walls of the guard cells [21]. This determines alterations in photosynthetic efficiency and gas exchange [22]. Leaf-level processes, such as transpiration, photosynthesis, and stomatal closure, are negatively influenced by salinity stress [23]. A decrease in stomatal conductance, photosynthesis, and efficiency of photosystem II (PSII) was indeed observed [24]. The effects of salinity on photosynthesis are directly attributed to stomatal limitations in the diffusion of gases, which alter photosynthesis and mesophyll metabolism [25].

This salt stress determines alterations in the leaf color, thickening of the leaf blade, which can become leathery [26,27], and the presence of necrosis and burns, especially along the leaf margins, due to the direct action of sodium ions that accumulate in the mesophyll [28]. Premature defoliation, deterioration of buds and stems, and a reduction in sprout growth can also be observed. The most sensitive plants can manifest metabolic alterations, such as an increase in stomatal resistance and resistance to water movement within the tissues [29,30].

In recent years, due to the increasing presence of residential or tourist structures in coastal areas [4], ornamental green spaces have spread to improve the aesthetic appearance of these areas and their possible use. In this context, selecting plants tolerant to salt aerosol [31] is relevant. Identifying species or cultivars less influenced by salinity [32] is a key aspect, together with functional irrigation management, in developing sustainable ornamental plant areas in coastal areas. It is essential to gain more knowledge about the morphological and physiological responses of plants under adverse environmental conditions [33], in light of selecting the most suitable plant species in salt spray conditions without compromising the aesthetic value. The large number of plant species used (or usable) for ornamental purposes [34] allows us to select from many suitable genotypes for different situations. Shrubs, especially those native to the Mediterranean region, appear suitable due to their rusticity and ability to adapt to the most diverse environmental conditions [35].

Identification of tolerant genotypes is, however, expensive due to the wide biodiversity characteristic of ornamental plants and due to the difficulty in identifying parameters suitable for defining responses to biotic and abiotic stresses [31,36,37,38]. A large number of species can potentially be used in green areas along the coast, but tolerance to marine aerosols is different among species [39] and sometimes among different cultivars [32].

A strategy to identify plants more resistant to saline aerosol can be useful to meet the demand for their use in tourist areas, both public and private, located along coastal areas as well as to decorate roads, flower beds, hotels, and private houses [4].

Numerous woody species used along roads, both in public recreation areas and car parking areas, are often selected only based on their aesthetic quality (flowers, bark, fruits, color of flowers), and there is no information available on their tolerance to saline aerosol [40].

Therefore, there is a need to identify easy-to-use and reliable salt stress indicators. These easy-to-use methodologies are handy tools to understand the intrinsic ability of an individual species to quickly adjust its metabolism under salt stress conditions.

Defining parameters that are easy to use and reliable can help in the difficult phase of choosing plant species. For ornamental purposes, the visual characteristics and, in particular, the percentage of leaf surface damaged by the toxic action of ions [41] are of great interest. Since leaves are the first target of salt spray damage, chlorophyll *a* fluorescence can be used as a tool to identify tolerant species. Measuring chlorophyll *a* fluorescence is non-destructive, fast, and easy to perform [42]. Gas exchange parameters could also be useful because they can be measured non-destructively.

In this context, this study aimed to identify plant response-based parameters suitable as markers for salt aerosol stress tolerance in ornamental shrubs. Such markers would be invaluable in prescreening suitable ornamental plants for landscaping near the sea. The second goal was to compare ornamental plant variations in response to salt spray to identify which species better adapts to salt stress conditions.

## 2. Materials and Methods

### 2.1. Plant Material and Experimental Procedures

This study was conducted in a greenhouse located near Catania, Italy (37°41′ N 15°11′ E 90 m a.s.l.). Twenty-four plant species were identified on the basis of our previous surveys (not published), in which we analyzed both the presence of ornamental plants in green spaces along coastal areas and the plants more easily available in the ornamental plant nurseries of the territory (Table 1). Shrubs were mainly chosen because of their wide use in the construction of hedges along urban coastal areas and, therefore, they are greater exposed to the effects of saline aerosol. Most of the species were native to the Mediterranean or Mediterranean-like environments; 50% of the species had sclerophyllous leaves or leaves characterized by structures (e.g., hairs or roughness) that allowed them to be suitable for resisting saline stress. Two-month-old rooted cuttings were transplanted into 2.7 L pots with a mixture of peat (60%), pumice and expanded clay (20%), field soil (10%), and composted horse manure (10%) and were fertilized with 2 g/L of Osmocote Plus (14:13:13 N,P,K + microelements). The plants were watered daily using a drip system with one emitter per plant (2 L h^−1^ each). The experiment started in September 2019; treatment was performed by spraying the crowns of plants with distilled water (control) and with seawater solutions twice a week for 60 days (until November 2019). The composition of simulated seawater solution was as follows: NaCl, Na_2_SO_4_, MgCl_2_, CaCl_2_, and KCl at concentrations of 23.48, 3.92, 4.98, 1.10, and 0.66 g L^−1^, with a concentration of 401.8 mM NaCl [5]. The mean air temperatures and relative humidity levels during the experimental periods were recorded on a data logger (CR 1000; Campbell Scientific Ltd., Loughborough, UK). The maximum and minimum temperatures were 18.4 and 27.4 °C, respectively, and the mean relative humidity levels ranged from 96.3 to 96.8%. The light intensity ranged from 4.6 to 15.5 MJ m^−2^ d^−1^.

### 2.2. Plant Growth Analysis

At the end of the experiment, nine pots per treatment (three per replication) were randomly chosen to measure the fresh and dry weights of shoots and roots, leaf area, leaf number, leaf damage, and specific leaf area. Plants located along the borders of blocks were not harvested. The dry weights (DWs) were obtained by drying the samples in a thermos-ventilated oven at 70 °C to a constant weight. The leaf areas were determined using a leaf area meter (Delta-T Devices Ltd., Cambridge, UK), and the leaf damage was determined as a percentage of the total area. The specific leaf area (SLA) was calculated as the ratio of the leaf area to the leaf dry weight.

### 2.3. Chlorophyll Content, Photosynthesis, and Chlorophyll a Fluorescence

At the end of the trial, the chlorophyll content was determined according to Moran and Porath [43]. At the beginning and every 20 days [T1 (0 days), T2 (20 days), T3 (40 days), T4 (60 days)] of the experimental period, the net photosynthetic rate (AN) was measured on fully expanded leaves using a CO_2_/H_2_O IRGA (LCi, ADC Bioscientific Ltd., Hoddesdon, UK). The measurements were carried out in clear conditions from 10:00 to 14:00 (solar time). After measuring photosynthesis, in the same leaves, chlorophyll *a* fluorescence was measured using a modulated chlorophyll fluorimeter OS1-FL (Opti-Sciences Corporation, Tyngsboro, MA, USA). Each leaf was dark-adapted for 20 min using cuvette clips supplied by the company. Fluorescence was expressed as the Fv/Fm ratio, which indicates the maximum quantum yield of PSII, where F0 = the minimum fluorescence, Fm = the maximum fluorescence of the dark-adapted state, and Fv = the variable fluorescence [44].

### 2.4. Statistics

**Figure 1:**
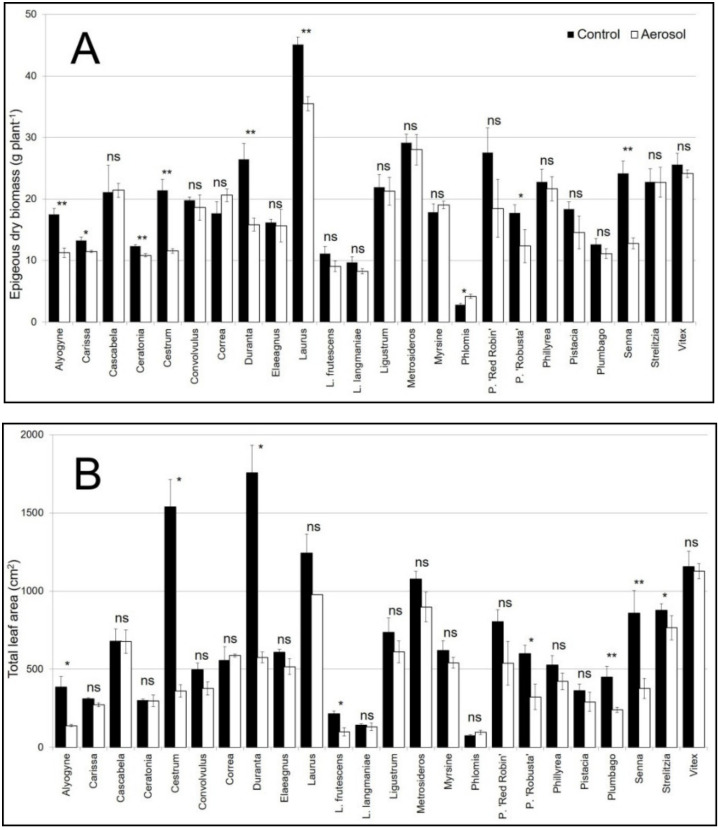
Epigeous dry biomass (**A**) and total leaf area (**B**) in twenty-four species of ornamental shrubs exposed to salt spray for 60 days. The vertical bars represent the S.E. of the means. Data were subjected to one-way ANOVA, and differences among means were determined for each species using Tukey’s post-test (*p* ≤ 0.05); n.s. not significant; * significant at *p* < 0.05; ** significant at *p* < 0.01.

**Figure 2:**
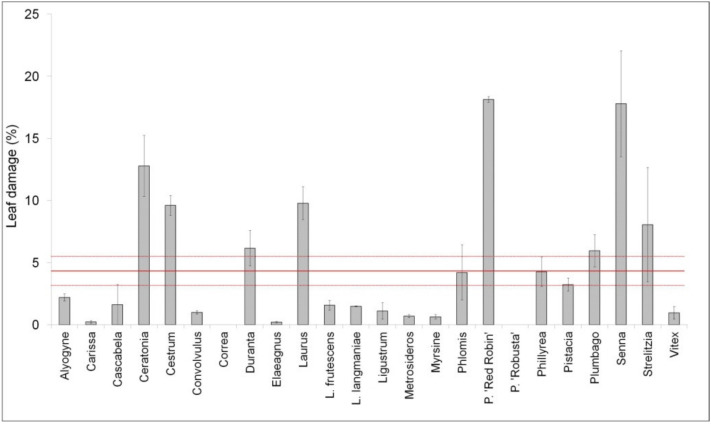
Leaf damage percentage in twenty-four ornamental species exposed to salt spray for 60 days. Values are means ± standard errors (*n* = 6). The horizontal red lines show the average value ± standard errors.

**Figure 3:**
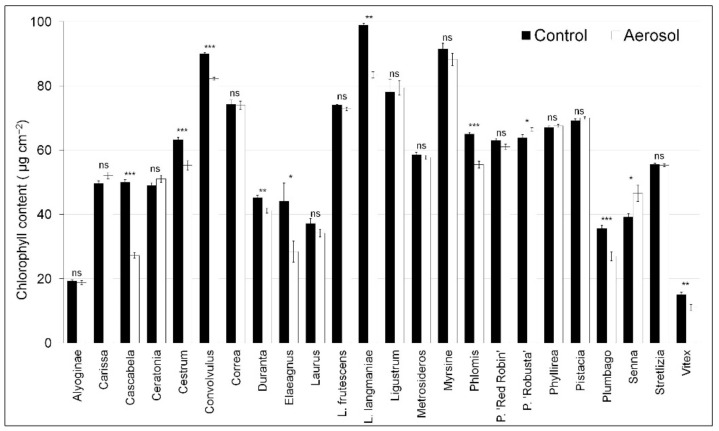
Chlorophyll content in twenty-four species of ornamental shrubs exposed to salt spray for 60 days. The vertical bars represent the S.E. of the means. Data were subjected to one-way ANOVA, and differences among means were determined for each species using Tukey’s post-test (*p* ≤ 0.05); n.s. not significant; * significant at *p* < 0.05; ** significant at *p* < 0.01; *** significant at *p* < 0.001.

The experiment was conducted in a randomized complete design with three replicates (the species were randomized within the treatment). Three replicates of 6 plants each (36 plants in total per species, 18 for each treatment) were used. The means were subjected to two-way variance (ANOVA) to determine the effects of aerosol, compared to the control plant, for each species at the end of the experiment. The data presented in Figure 1, Figure 2 and Figure 3 are the means ± SE. Data were subjected to one-way ANOVA, and differences among means were determined for each species using Tukey’s post-test (*p* ≤ 0.05). HeatMaps were generated with MS excel using conditional formatting rules, and statistical differences were highlighted using asterisks (*p* < 0.05).

## 3. Results

Shrub responses to the salt spray were species-dependent. Significant interactions between species and treatments were observed for the shoot and total dry biomass (Table 2). Compared to the control, notable shoot dry weight reductions were observed in Cestrum (−46%), Alyogyne (−36%), Senna (−47%), Duranta (−40%), and in P. ‘Robusta’ (−30%). Conversely, an increase was observed in Phlomis (+30%). In all other species, no significant difference was observed (Figure 1A). The reduction in total dry biomass was similar for all species (Figure S1A). The Root-to-Shoot ratio was significantly affected only by species (Table 2). Significant interactions between species and treatments were also observed for total leaf area and leaf number (Table 2). A decrease in leaf area was observed in several species: Cestrum (76%), Duranta (67%), Alyogyne (64%), L. frutescens (54%), P. ‘Robusta’, and Plumbago (46%) (Figure 1B). This variation was related to a reduction in leaf number (Figure S1B). A similar response was observed for SLA; all species showed a reduction in this parameter due to the effect of salt spray. Only two species, Ceratonia and Senna, showed an increase in this parameter (25 and 46%, respectively) (Figure S1C).

The percentage of leaf damage was significantly different among species. The average percentage of leaf damage was 5%. Among species, the maximum value (~18%) was observed in P. ‘Red Robin’ and ‘Senna’; no leaf damage was observed in Correa and P. ‘Robusta’ (Figure 2).

The chlorophyll content was significantly different for some species (Figure 3). Among the shrubs, large reductions were observed in Cascabela (45%), Elaeagnus (35%), and L. langmanie (16%).

**Figure 4:**
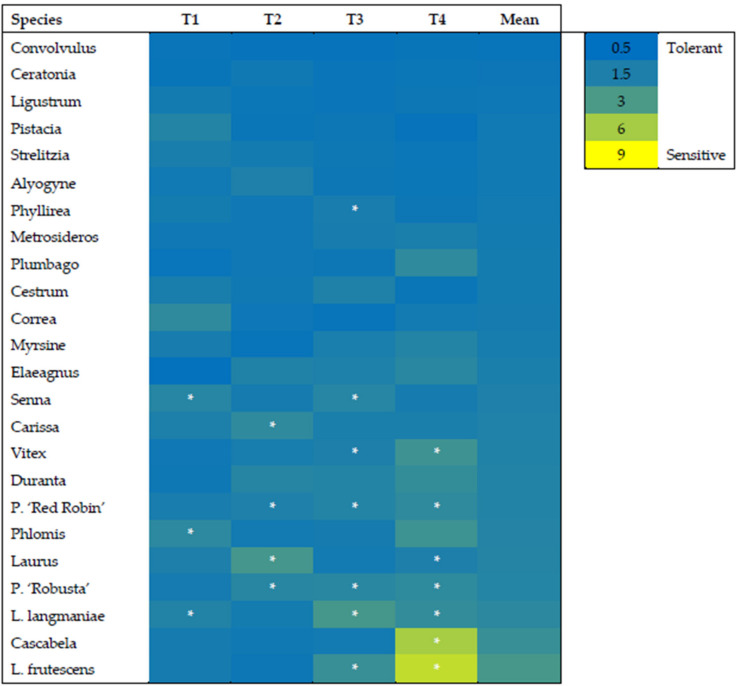
HeatMap of the Net photosynthesis (AN) ratio between control and aerosol treatments in twenty-four species of ornamental shrubs exposed to salt spray. Plants were subjected twice a week to nebulization treatment with simulated seawater solution for 60 days. At the beginning and every 20 days [T1 (0 days), T2 (20 days), T3 (40 days), T4 (60 days)] of the experimental period, the ratio was calculated. The asterisks depict statistically significant differences between control and treated plants for each species as determined by Tukey’s test (*p* < 0.05).

The Net Photosynthesis (A_N_) varied among species, treatments, and time points. The highest value was found in Phlomis control T1 (11.46 µmol m^−2^ s^−1^) and the lowest in stressed Cascabela T4 (0.67 µmol m^−2^ s^−1^). The A_N_ declined in sensitive species at T4, and, at this time point, differences among species were visible (Figure S2, Figure 4).

Among the different species, there were few that showed A_N_ reductions, even in control plants, including Cestrum, Phlomis, P. ‘Red Robin’, P. ‘Robusta’, Senna, and Vitex. This decline was observed at T3 or T4 and might be associated with higher-temperature stress.

**Figure 5:**
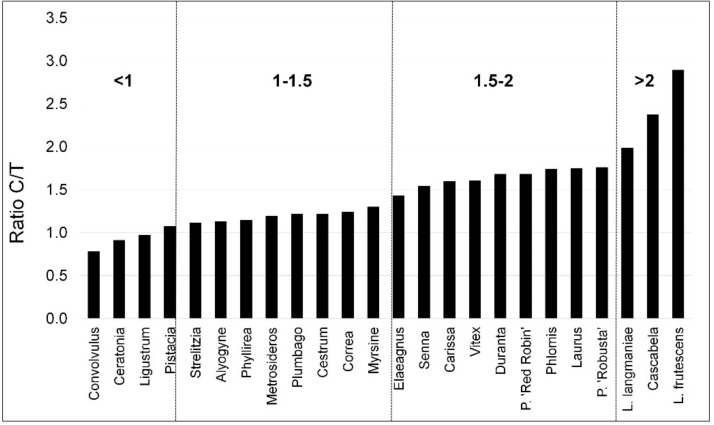
Distribution of the different studies species in four groups of tolerance, C (Control) and T (salt spray treatment) ratio. Below 1 indicates more tolerant species and above 2 indicates more sensitive ones.

The HeatMap of the ratio between Control and Aerosol treatment revealed that the most sensitive species were L. langmaniae, Cascabela, and L. frutescens (Figure 4), with ratios all above 2. On the contrary, the most tolerant were Convolvulus, Ceratonia, and Ligustrum, which showed ratios below 1. These results indicated that A_N_ was even higher in stressed plants, considering the mean of the different time points. Two intermediate groups could be identified, with ratios from 1 to 1.5 and 1.5 to 2. The first group from 1 to 1.5 included Pistacia, Strelitzia, Alyogyne, Phyllirea, Metrosideros, Plumbago, Cestrum, Correa, Myrsine, and Elaeagnus. The second group with ratios from 1.5 to 2 included Senna, Carissa, Vitex, Duranta, P. ‘Red Robin’, Phlomis, Laurus, and P. ‘Robusta’ (Figure 5).

**Figure 6:**
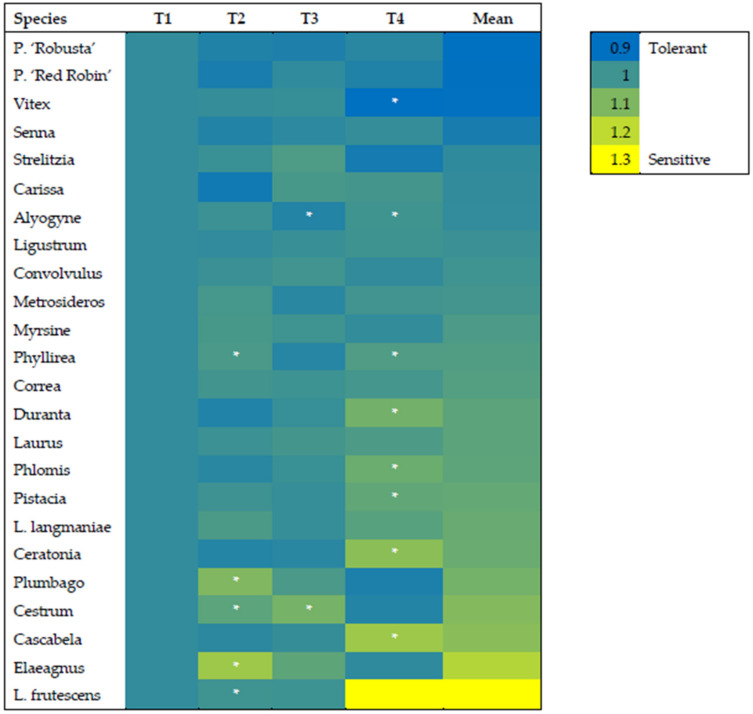
HeatMap of the maximum quantum efficiency of photosystem II (Fv/Fm ratio) between control and treatment groups for twenty-four species of ornamental shrubs exposed to salt spray. Plants were subjected twice a week to nebulization treatment with simulated seawater solution for 60 days. At the beginning and every 20 days [T1 (0 days), T2 (20 days), T3 (40 days), T4 (60 days)] of the experimental period, the ratio was calculated. The asterisks depict statistically significant differences between control and treated plants for each species as determined by Tukey’s test (*p* < 0.05).

Chlorophyll *a* fluorescence is a good non-destructive marker of stress in plants. In our experiment, the maximum quantum efficiency of PSII (Fv/Fm ratio) declined in several species, especially after T4, but this reduction did not correspond to a reduction in A_N_. The Fv/Fm ratio ranged from 0.99 to 0.46. The lowest value was found for L. frutescens exposed to marine aerosol at T4 (Figure 6). Significant reductions in the Fv/Fm ratio were observed at T4 for Cascabela, Ceratonia, Duranta, Phlomis, Phyllirea, Pistacia, and Vitex. The Fv/Fm ratio also declined in the control, proceeding with the time points. This indicated the environmental conditions affected the stress status of the plants.

Pearson’s correlation coefficients are shown in Table 3. Overall, 136 correlations were analyzed, of which 72 (60% of the total) showed significance (60 positive and 12 negative relationships). The net photosynthesis showed a strong, positive correlation with all morphological parameters (0.311 *** between A_N_ and ULA), with only the leaf number being negatively correlated (−0.181 *).

In the case of chlorophyll *a* fluorescence variables, stronger, positive correlations were found between Fm and EDM (0.287 ***), Fm and A_N_ (0.380 ***), Fm and F0 (0.632 ***), Fv and LDM (0.366 ***), Fv and EDM (0.305 ***), Fv and A_N_ (0.417 ***), Fv and F0 (0.447 ***), Fv and Fm (0.976 ***), Fv/Fm and A_N_ (0.345 ***), Fv/Fm and F0 (0.476 ***), Fv/Fm and Fm (0.354 ***), and between Fv/Fm and Fv (0.542 ***) (Table 3). Strong inverse correlations were observed between LN and RDM (−0.196 **), LN and R/S (−0.357 ***), LN and ULA (−0.351 ***), F0 and R/S (−0.296 ***), Fm and R/S (0.344 ***), and Fv and R/S (−0.313 ***) (Table 3).

## 4. Discussion

Plants grown along coastal areas are frequently exposed to salt spray. The identification of plants tolerant to marine aerosol is particularly important for shrubs and trees that will be planted in green ornamental areas. Non-destructive methods for fast and efficient screening are needed at the nursery level. In this study, morpho-physiological parameters were selected as salt spray salinity markers and were tested for a wide number of ornamental plants. The response of ornamental shrubs to salt stress appeared different among the species analyzed. On the basis of the ornamental plant responses, the best parameters have been suggested. In urban and peri-urban coastal areas, the selection of ornamental plants tolerant to salt stress is very important, and limited information is available in the literature to assist [5]. Regarding the correct selection of plants resistant to salt aerosol, it is important to understand the morphological and/or physiological responses of plant species to this stress. Our results showed there were differences in the responses of ornamental shrubs; salt spray had different effects on biomass distribution according to the species. The first modifications observed as a consequence of stress were related to the epigeous portion of the plant; indeed, salinity reduces cellular division and growth [45]. Growth reduction, in fact, is a typical response to salt stress in more sensitive species [46]. Among the species, notable total dry weight reductions were observed in Cestrum, Alyogyne, Senna, Duranta, and P. ‘Robusta’. Conversely, an increase was observed in Phlomis. Different authors have shown that plant growth can be inhibited by salt spray, and this inhibition often is due to reductions in the leaf area [47,48,49]. Decreased shoot growth is a common response to salt and water stress, resulting in a reduction in new leaf emissions and leaf growth [50]. In our study, in the species that had a reduced epigeous dry biomass, a reduction in the leaf surface area was observed. This reduction leads to decreases in area for water loss through transpiration and changes in water use efficiency in plants, which have been identified to be adaptive mechanisms [51].

The leaf area impacts a plant’s strategy to adapt to environmental change, and it is also connected to plant growth, light interception, gas exchange, and ornamental value [52].

Furthermore, the effects of stress are also exerted on the chlorophyll content of the leaves. Cha-um et al. [53], in a study of 13 rice cultivars subjected to salt stress, reported chlorophyll accumulation was dissimilar between salt-tolerant and salt-sensitive cultivars. Similarly, in our study, the salt-sensitive species showed reduced chlorophyll content.

In different species, exposure to a progressive increase in salinity induces an increase in the chlorophyll concentration until it reaches a maximum value, and then it declines. This increase in chlorophyll is due to adaptation, and the subsequent decrease is due to lack of tolerance to high salinity above the sensitivity threshold of the species. The reduction in chlorophyll content is due to inhibited chlorophyll turnover resulting from salinity accumulating in the cytoplasm and blocking enzyme activities [54,55]. The increase in salt and membrane disruption can lead to necrosis of leaves. Net photosynthesis is correlated to almost all morphological parameters concerning the leaf apparatus. Although, the highest correlation was found with ULA. This result can be explained considering the mechanism of saline aerosol damage on leaves.

In ornamental plants, the response to stress cannot only be measured in terms of growth [56]; in fact, it is often linked more to the visual appearance than to the growth or biomass reduction in stressful conditions. The growth rate and/or accumulation of dry biomass are parameters that are commonly used to evaluate the tolerance of plants to salt, but a destructive survey must be carried out. For these reasons, numerous studies have evaluated the response of ornamental plants to salt stress using visual methods [13,57,58]. Almost all shrubs analyzed in our study developed necrosis when exposed to salt spray, demonstrating that salt penetrates the leaves through the cuticle [59], through breakages in the cuticle [1,60], or the stomata [61,62,63]. However, the percentages of leaf surface necrosis were significantly different among species. Compared to the average value (~5%), some genotypes had much higher damage rates, reaching the maximum value (18%) in Cassia and P. ‘Red Robin’; conversely, no damage was observed in two shrubs, Correa and P. ‘Robusta’. One of the consequences of necrotic area formation is a reduction in photosynthetic activity. Necrotic spots in *Pinus rigida* seedlings were reported to decrease net photosynthesis and ultimately led to reduced growth and long-term survivorship [64]. Other species showed leaf damage due to salt spray: *Solidago puberula*, *S. rugosa*, *Gaylussacia baccata*, and *Quercus ilicifolia* [65].

Photosynthesis is the most important physiological process in plants, and it must remain active, even under stress conditions, to maintain the growth and survival of the species. Plants under suboptimal conditions induce several biochemical and physiological strategies to cope with the adverse environment [34]. Therefore, the decline in photosynthesis activity represents an extreme mechanism for plants to avoid cell damage. Under abiotic stresses such as drought, salinity, high temperature, etc., the light use efficiency of leaves declines to avoid absorbing an excess of energy that can impair the photosystems and the photosynthetic apparatus [66,67]. Salinity stress in plants induces phytotoxicity as well as drought stress-like effects. Sodium and chloride in plants must be stored in vacuoles or old leaves, and specific neutral or compatible solutes as well as protective molecules accumulate to protect the cell functionality [68]. Therefore, the result of this study suggests a close relationship between the epigeous portion and leaf functionality.

Plants used in green areas exposed to stress conditions can lose their ornamental quality and have negative effects on the visual appearance of urban or peri-urban areas. Salinity is a common problem in coastal areas that affects the vegetation of the landscape. There is increased interest to identify different physiological and biochemical parameters at the nursery level that can be used as markers to select tolerant species [69]. Good photosynthesis activity, even under salinity aerosol stress, can contribute to the biosynthesis of different related primary metabolism antioxidant defense mechanisms, which help plants in detoxifying free radicals induced by stress conditions.

Chlorophyll *a* fluorescence has been used to evaluate the saline aerosol tolerance in several species that can be potentially used as ornamental plants in coastal areas [4]. The most tolerant species did not show a decline in the chlorophyll *a* fluorescence induction curve, while the most sensitive showed a strong reduction in the measured parameters. Tolerance was also associated with leaf morphology and structure. The maximum quantum efficiency of photosystem II (Fv/Fm ratio) usually declined just after the photosynthesis activity reduced, and the performance index (PI) was the most sensitive parameter [4]. These results have been observed in a wide range of ornamental species exposed to salinity stress [5]. At the eco-physiological level, the most sensitive species to aerosol salinity show reductions in the photosynthesis activity and Fv/Fm ratio. The intensity of the decline is also affected by the temperature of the environment. During summer the stress intensity increases, and if plants reduce the gas exchange, there is a reduction in transpiration and increase in leaf temperature associated with several physiological disorders such as leaf abscission and chlorophyll loss [70,71]. In our study, the photosynthesis activity widely varied among species, and this could be exploited to identify the most suitable species for environments or coastal areas with marine aerosol problems. The Fv/Fm ratio showed smaller variations, which can be associated with the severe stress conditions in plants.

Pearson’s correlation analysis revealed a close relationship between light use efficiency parameters (photosynthesis and chlorophyll *a* fluorescence) and leaf area biomass accumulation or root/shoot ratio. These significant results demonstrated how adaptation-associated parameters were well correlated and can potentially be used to estimate the intensity response of the plants to salt spray. A weaker correlation was observed between photosynthesis and leaf damage because the gas exchange and chlorophyll *a* fluorescence measurements were taken on less damaged leaves. However, these results are not contradictory to the aim of the work because the selected parameters were highly correlated with tolerance traits and not with plant sensitivity to salt spray.

The overall data analysis showed that the physiological and biochemical parameters can represent good markers for selecting ornamental plants that can be grown in seaside areas.

Morphological traits are also good indicators of the stress effect intensity, but they cannot be determined without using destructive analyses, especially for root-related parameters. Among the different parameters studied, net photosynthesis was most strongly associated to salt spray stress tolerance. Different from chlorophyll *a* fluorescence parameter, gas exchange data effectively characterized the response and adaptation degree to stressful environments.

The A_N_ can be used at the nursery level to select ornamental species that will be suitable in areas exposed to marine aerosol without compromising the visual appeal of the area. Moreover, this is a non-destructive method that can also be used to monitor the adaptation of plants over time.

Plant survival is not sufficient to maintain the ornamental value of the landscape because the quality of green areas depends on the leaf turnover, leaf abscission, leaf yellowing, and chlorophyll concentration. A combined evaluation of these parameters can help in the species selection.

## 5. Conclusions

Photosynthesis activity and related parameters can be used in a non-destructive screening method to identify plant species tolerant to saline aerosol. However, physiological and biochemical parameters alone do not ensure a plant’s high aesthetical quality; this is mainly represented by the leaf color and vitality. In tourist areas, ornamental plants must also be high quality in terms of visual appearance. Therefore, the physiological and biochemical parameters measured must also incorporate high-quality leaf color and the absence, or very low levels, of leaf necrosis.

Following appropriate procedures and protocols, this method can be used at the nursery level to cluster ornamental species for use as implants in urban and peri-urban green areas located along the coast.

## Figures and Tables

**Table 1:** Main botanical information for the twenty-four ornamental shrubs involved in this study.

Plant Name ^1^	Latin Name	Family	Cultivar	Origin	Main Ornamental Organs	Leaf
Alyogyne	*Alyogyne huegelii* (Endl.) Fryxell	Malvaceae		Australia	flowers	Non-sclerophyllous
Carissa	*Carissa macrocarpa* (Eckl.) A.DC.	Apocynaceae		South Africa	flowers and fruits	Sclerophyllous
Cascabela	*Cascabela thevetia* (L.) Lippold	Apocynaceae		Mexico	flowers	Non-sclerophyllous
Ceratonia	*Ceratonia siliqua* L.	Leguminosae		Mediterranean region	leaves	Sclerophyllous
Cestrum	*Cestrum fasciculatum* (Schltdl.) Miers	Solanaceae	‘Newelli’	Mexico	flowers	Non-sclerophyllous
Convolvulus	*Convolvulus cneorum* L.	Convolvulaceae		Mediterranean region	flowers	Non-sclerophyllous
Correa	*Correa alba* Andrews	Rutaceae		Australia	leaves and flowers	Non-sclerophyllous
Duranta	*Duranta erecta* L.	Verbenaceae		Mexico	flowers and fruits	Non-sclerophyllous
Elaeagnus	*Elaeagnus × submacrophylla* Servett.	Elaeagnaceae		China, Japan	leaves	Sclerophyllous
Laurus	*Laurus nobilis* L.	Lauraceae		Mediterranean region	leaves	Sclerophyllous
L. frutescens	*Leucophyllum frutescens* (Berland.) I.M. Johnst.	Scrophulariaceae		Texas and Mexico	flowers	Non-sclerophyllous
L. langmaniae	*Leucophyllum langmaniae* Flyr	Scrophulariaceae		Mexico	flowers	Non-sclerophyllous
Ligustrum	*Ligustrum japonicum* Thunb.	Oleaceae		Eastern Asia	leaves	Sclerophyllous
Metrosideros	*Metrosideros robusta* A.Cunn.	Myrtaceae	‘Thomasii’	New Zealand	leaves	Sclerophyllous
Myrsine	*Myrsine africana* L.	Primulaceae		Africa	leaves	Sclerophyllous
Phlomis	*Phlomis fruticosa* L.	Lamiaceae		Mediterranean region	flowers	Non-sclerophyllous
P. ‘Red Robin’	*Photinia × fraseri* Dress	Rosaceae	‘Red Robin’	Only cultivated	leaves	Sclerophyllous
P. ‘Robusta’	*Photinia × fraseri* Dress	Rosaceae	‘Robusta Compacta’	Only cultivated	leaves	Sclerophyllous
Phillyrea	*Phillyrea angustifolia* L.	Oleaceae		Mediterranean region	leaves	Sclerophyllous
Pistacia	*Pistacia lentiscus* L.	Anacardiaceae		Mediterranean region	flowers	Sclerophyllous
Plumbago	*Plumbago auriculata* Lam.	Plumbaginaceae		South Africa	flowers	Non-sclerophyllous
Senna	*Senna corymbosa* (Lam.) H.S.Irwin & Barneby	Leguminosae		Southern America	flowers	Non-sclerophyllous
Strelitzia	*Strelitzia reginae* Banks	Strelitziaceae		South Africa	flowers	Sclerophyllous
Vitex	*Vitex triflora* Vahl	Lamiaceae		Southern America	flowers	Non-sclerophyllous

^1^ Names used in the text to indicate the different genotypes.

**Table 2:** F-values related to the main factors and their interaction between the observed and calculated variables, with significance determined from ANOVA.

Parameters	Species (S)	Treatment (T)	S X T
Total dry biomass	58.5 ***	34.6 ***	2.7 ***
Epigeous dry biomass	33.2 ***	36.2 ***	2.6 **
Root/Shoot	21.5 ***	1.4 n.s.	1.6 n.s.
Total leaf area (cm^2^)	16.6 ***	44.1 ***	4.1 ***
Leaf number (n plant^−1^)	156.8 ***	34.4 ***	3.0 ***

n.s.: not significant; **, *** significant at *p* < 0.01 and 0.001, respectively.

**Table 3:** Relationship between morphological and physiological parameters of ornamental shrubs under salt stress treatment.

	SDM	LDM	RDM	EDM	TDM	R/S	LA	ULA	LN	SLA	LD	A_N_	F0	F_M_	F_V_
LDM	0.300 ***														
RDM	0.492 ***	0.629 ***													
EDM	0.695 ***	0.894 ***	0.705 ***												
TDM	0.644 ***	0.827 ***	0.921 ***	0.925 ***											
R/S	0.091ns	0.055 *	0.699 ***	0.084ns	0.420 ***										
LA	0.425 ***	0.734 ***	0.554 ***	0.753 ***	0.709 ***	0.181 *									
ULA	−0.123ns	0.244 **	0.420 ***	0.126ns	0.294 ***	0.510 ***	0.353 ***								
LN	0.032ns	0.005ns	−0.196 **	0.019ns	−0.094ns	−0.375 ***	0.011ns	−0.351 ***							
SLA	0.268 ***	0.092ns	0.221 **	0.193*	0.224 **	0.264 ***	0.611 ***	0.455 ***	−0.027ns						
LD	0.013ns	−0.138ns	0.215 **	−0.098ns	0.061ns	0.407 ***	−0.171 *	0.206 *	−0.216**	−0.062ns					
A_N_	−0.013ns	0.387 ***	0.244 **	0.286 ***	0.287 ***	0.171 *	0.228 **	0.311 ***	−0.181 *	0.037ns	−0.035ns				
F0	−0.095ns	0.183 *	−0.092ns	0.093ns	0.002ns	−0.296 ***	0.108ns	−0.026ns	0.251 **	−0.062ns	−0.107ns	0.076ns			
F_M_	0.030ns	0.362 ***	−0.010ns	0.287 ***	0.152ns	−0.344 ***	0.129ns	−0.020ns	0.141ns	−0.095ns	−0.183 *	0.380 ***	0.632 ***		
F_V_	0.061ns	0.366 ***	0.014ns	0.305 ***	0.175 *	−0.313 ***	0.118ns	−0.016ns	0.093n.s.	−0.093n.s.	−0.181 *	0.417 ***	0.447 ***	0.976 ***	
F_V_/F_M_	0.124ns	0.171*	0.101ns	0.187 *	0.156ns	−0.015ns	0.015ns	0.031ns	−0.123ns	−0.029ns	−0.075ns	0.345 ***	−0.476 ***	0.354 ***	0.542 ***

Significant levels at *p* < 0.05 are presented by *, at *p* < 0.01 are presented by **, and at *p* < 0.001 by *** using Pearson’s correlation coefficients. Legend: SDM = stem dry matter; LDM = leaf dry matter; RDM = root dry matter; EDM = epigeous dry matter; TDM = total dry matter; R/S = root/shoot ratio; LA = leaf area; ULA = unit leaf area; LN = leaf number; SLA = specific leaf area; LD = leaf damage; A_N_ = net photosynthesis; F0 = minimum fluorescence; F_M_ = maximal fluorescence; F_V_ = variable fluorescence; F_V_/F_M_ = maximal quantum yield of PSII. Cell contents: Pearson correlation coefficient and *p*-Value.

## Supplementary Materials

The following are available online at https://www.mdpi.com/2079-7737/9/9/250/s1, Figure S1: Total dry biomass (A), leaf number (B), and SLA (C) in twenty-four species of ornamental shrubs exposed to salt spray after 60 days; Figure S2: Net Photosynthesis (A_N_) and chlorophyll *a* fluorescence (Fv/Fm) in twenty-four species of ornamental shrubs exposed to aerosol marine.
