# Supplementary material for: An Evaluation of Different Parameters to Screen Ornamental Shrubs for Salt Spray Tolerance
Source: Biology (Basel). 2020 Aug 27;9(9):250. doi: 10.3390/biology9090250 (PMC7564769; doi:10.3390/biology9090250)
Supplement: Supplementary file 1 [file biology-09-00250-s001.pdf]

biology

Supplementary material

An Evaluation of Different Parameters to Screen Ornamental Shrubs for Salt Spray Tolerance

Stefania Toscano, Ferdinando Branca, Daniela Romano and Antonio Ferrante

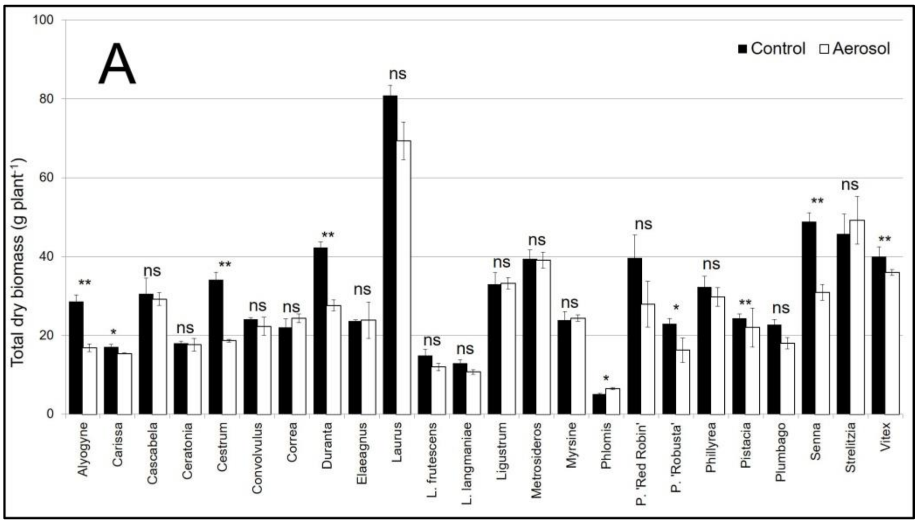

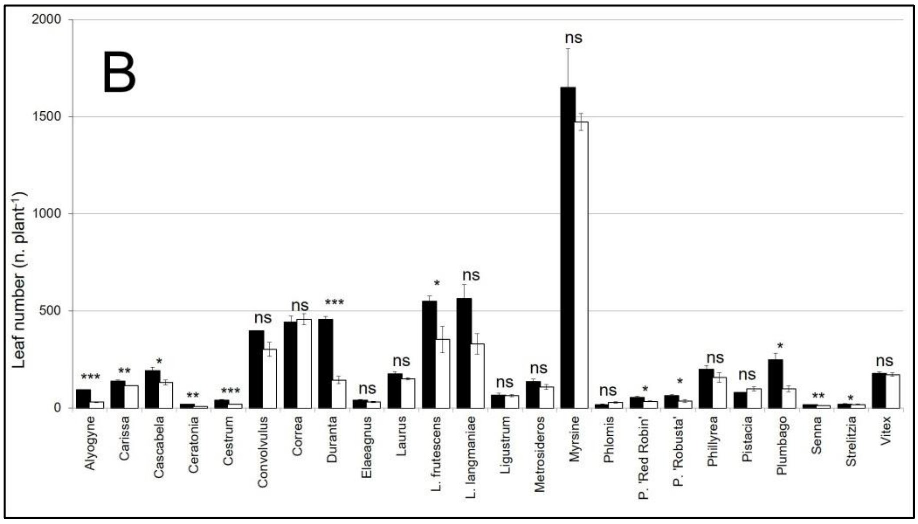

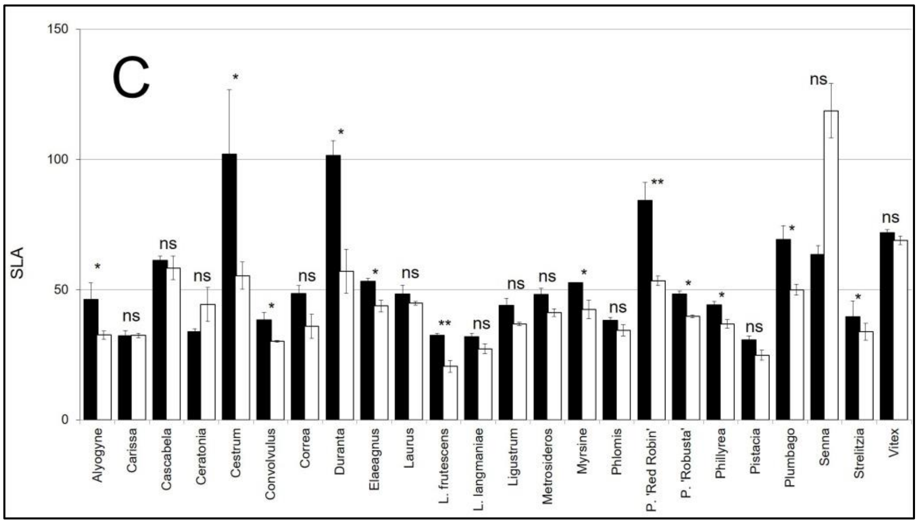

Figure S1. Total dry biomass (**A**), leaf number (**B**), and SLA (**C**) in twenty-four species of ornamental shrubs exposed to salt spray after 60 days. The vertical bars represent the S.E. of the means. Data were subjected to one-way ANOVA and differences among means were determined for each species using Tukey's post-test (P ≤ 0.05). n.s. not significant; * significant at p < 0.05; ** significant at p < 0.01; *** significant at p < 0.001.

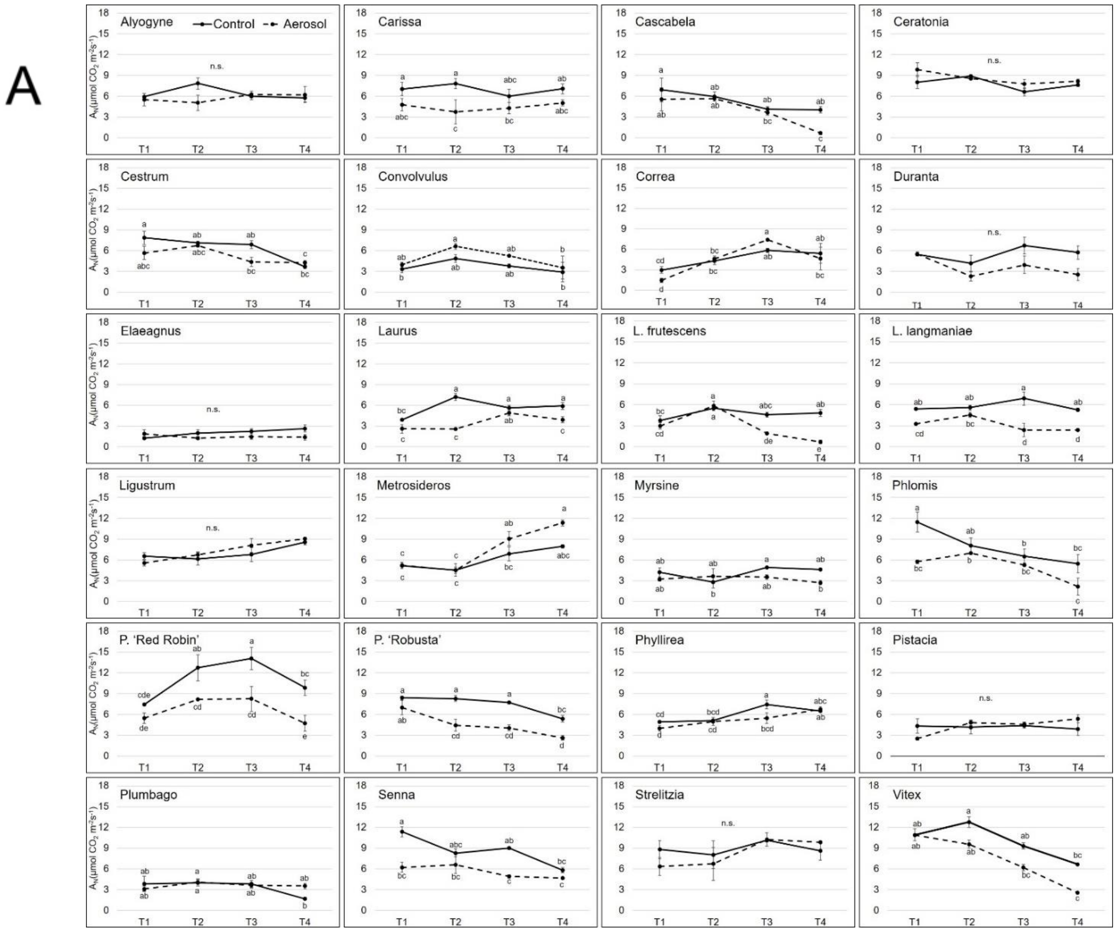

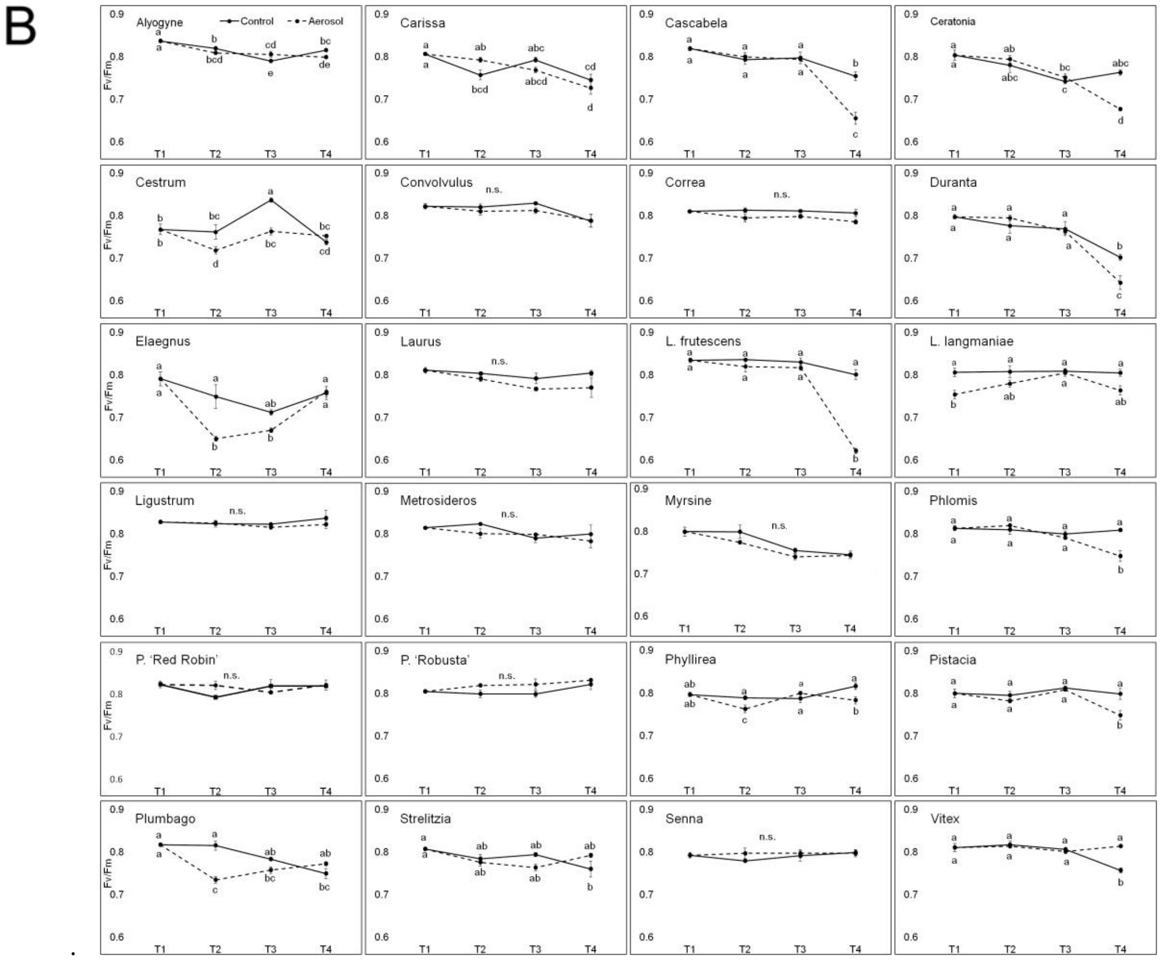

Figure S2. Net Photosynthesis (A_N) and chlorophyll *a* fluorescence (Fv/Fm) in twenty-four species of ornamental shrubs exposed to aerosol marine. **(A)** Net Photosynthesis (A_N) in twenty-four species of ornamental shrubs exposed to aerosol marine. Plants were subjected to twice a week nebulization treatment with simulated seawater solution for 60 days. At the beginning and every 20 days [T1 (0 days), T2 (20 days), T3 (40 days), T4 (60 days)] of the experimental period, the Net Photosynthesis (AN) was measured. The vertical bars represent the S.E. of the means. Their absence indicates that the size was less than the symbol. The letters depict statistically significant differences between control and treated plants for each species as determined by Tukey's test ($p < 0.05$). **(B)** Chlorophyll a fluorescence (Fv/Fm) in twenty-four species of ornamental shrubs exposed to aerosol marine. Plants were subjected to twice a week nebulization treatment with simulated seawater solution for 60 days. At the beginning and every 20 days [T1 (0 days), T2 (20 days), T3 (40 days), T4 (60 days)] of the experimental period, the Chlorophyll a fluorescence (Fv/Fm) was measured. The vertical bars represent the S.E. of the means. Their absence indicates that the size was less than the symbol. The letters depict statistically significant differences between control and treated plants for each species as determined by Tukey's test ($p < 0.05$).

© 2020 by the authors. Licensee MDPI, Basel, Switzerland. This article is an open access article distributed under the terms and conditions of the Creative Commons Attribution (CC BY) license (http://creativecommons.org/licenses/by/4.0/).
